# A Discussion on the Interpretation of the Darcy Equation in Case of Open-Cell Metal Foam Based on Numerical Simulations

**DOI:** 10.3390/ma9060409

**Published:** 2016-05-25

**Authors:** Sven De Schampheleire, Kathleen De Kerpel, Bernd Ameel, Peter De Jaeger, Ozer Bagci, Michel De Paepe

**Affiliations:** 1 Department of Flow, Heat and Combustion Mechanics, Ghent University, Sint-Pietersnieuwstraat 41, Ghent 9000, Belgium; Kathleen.DeKerpel@ugent.be (K.D.K.); Bernd.Ameel@ugent.be (B.A.); Ozer.Bagci@UGent.be (O.B.); michel.depaepe@ugent.be (M.D.P.); 2 NV Bekaert SA, Bekaertstraat 1, Zwevegem 8500, Belgium; Peter.DeJaeger@bekaert.com

**Keywords:** volume averaging theory, permeability, inertial coefficient, Darcy, Forchheimer, metal foam, pressure drop

## Abstract

It is long known that for high-velocity fluid flow in porous media, the relation between the pressure drop and the superficial velocity is not linear. Indeed, the classical Darcy law for shear stress dominated flow needs to be extended with a quadratic term, resulting in the empirical Darcy–Forchheimer model. Another approach is to simulate the foam numerically through the volume averaging technique. This leads to a natural separation of the total drag force into the contribution of the shear forces and the contribution of the pressure forces. Both representations of the total drag lead to the same result. The physical correspondence between both approaches is investigated in this work. The contribution of the viscous and pressure forces on the total drag is investigated using direct numerical simulations. Special attention is paid to the dependency on the velocity of these forces. The separation of the drag into its constituent terms on experimental grounds and for the volume average approach is unified. It is shown that the common approach to identify the linear term with the viscous forces and the quadratic term with the pressure forces is not correct.

## 1. Introduction

### 1.1. Open-Cell Metal Foam

There is a continuous search for new fin designs and materials. One of these designs is open cell-metal foam [1]. Despite the quite labor-intensive process of making open-cell metal foam, it has found its way to an increasing number of applications [2,3]. There are numerous types of open-cell metal foams, two of which are especially common. The first type is made through an investment casting process, usually based on a polyurethane preform. The second common type is based on a similar preform, but is made through an electrophoretic deposition process. To compare those two types of foam, not only the materials out of which they are made, but also the inner volumes of the strut are different. As a result of the casting process, the strut is solid, while the strut is hollow after the electrophoretic process. This has an impact on the thermal conductivity of the resulting foam material. Furthermore, in case of the casted manufacturing procedure, the polyurethane can first be waxed in order to increase the strut’s equivalent diameter [4]. This results in slightly different foam topologies compared to those of commercial foam samples [5].

**Figure 1:**
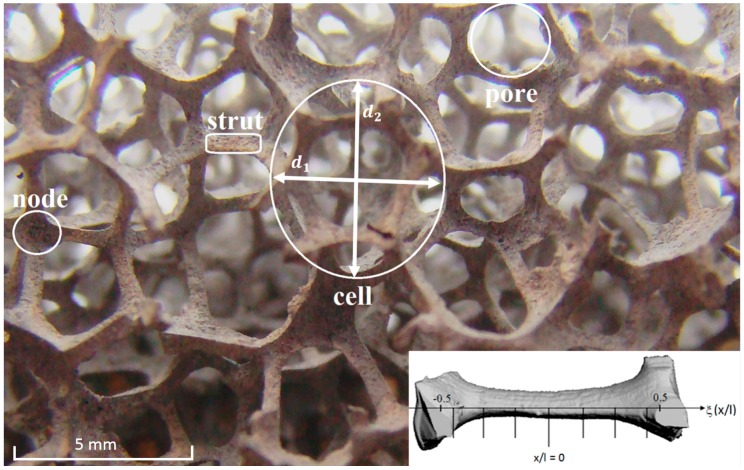
The nomenclature of an open-cell metal foam and its strut thickness variation.

The nomenclature of open-cell metal foam is illustrated in Figure 1. Struts interconnect the nodes and form both cells and pores. The shape of the struts themselves depends on the porosity. The thickness varies along the axial direction as illustrated in Figure 1. If the replication process is based on a polyurethane preform, then the cells are elongated in one direction ($d_{2}$ in Figure 1) due to the effect of gravity in manufacturing the preform [6]. Designing new applications with foam requires a profound knowledge of both the heat transfer coefficient and the flow resistance [7,8]. In this paper, only the flow resistance will be discussed.

### 1.2. Determination of Flow Resistance in Open Literature

The creeping flow through porous media is described by the Darcy equation, which relates pressure drop to velocity (Equation (1)). In Equation (1), κ is the permeability of the porous medium. After the transitional regime, it is experimentally shown that the pressure drop becomes quadratic with velocity (Equation (2)). The equation to capture this behavior is called the Darcy–Forchheimer equation (or Hazen–Dupuit–Darcy). In this equation, β is known as the inertial coefficient. Both permeability and inertial coefficient are originally seen as material properties [9]. They are exclusively related to the structure of the porous medium.

(1) $$\nabla P=\frac{\mu }{\kappa }V$$

(2) $$\nabla P=\frac{\mu }{\kappa }V+\rho \beta V^{2}$$

Dukhan *et al.* [10] mentioned that the Darcy equation accounts for the *viscous drag* while the Forchheimer term ($\rho \beta V^{2}$) corresponds to the *form drag*. This is also linked with the generally known interpretation of the Reynolds number. The Reynolds number represents the ratio of the momentum flux to the viscous stress. For many (but not all) applications, this can also be interpreted as the ratio between the inertial forces and the viscous forces. For low Reynolds number, the viscous forces are dominant and for high Reynolds numbers the inertial forces will become dominant. It is expected that when viscous stress dominates, that the relation between pressure drop and velocity is linear. However, for higher flow velocities, the linear pressure drop does not follow the Darcy equation. Du Plessis and Woudberg [11] provided an expression for the critical Reynolds number for departure from the Darcy regime, which only depends on the porosity of the porous medium ($c_{d}$ in Equation (3) is 1.9).

(3) $$Re_{c}=\frac{50.8\;\phi \;{\left(1-\phi \right)}^{\frac{1}{3}}}{c_{d}\left[1-{\left(1-\phi \right)}^{\frac{1}{3}}\right]}$$

In contrast, authors like Zeng and Grigg [12] illustrated that rather a Forchheimer number (Equation (4)) should be used to define the departure from Darcy regime. This number is defined as the ratio of the pressure gradient to the viscous resistance. As the Forchheimer number is related to the non-Darcy effect, a non-Darcy effect of 10% gives a Forchheimer number of 0.11. This is called the critical Forchheimer number.

(4) $$Fo=\frac{\kappa \beta \rho V}{\mu }$$

In open literature, there is a large discrepancy in results for permeability and inertial factor. Antohe *et al.* [3] reported differences when using different working fluids. This clearly indicates that the permeability and loss factor do not just depend on the structure of the porous medium, but on the fluid as well. Bonnet *et al.* [13] has shown that for a given pore size, the pressure drop coefficients are dispersed over at least one order of magnitude for high flow rates ($Re_{d_{p}}>1000$), and over more than two orders of magnitude for low flow rates ($1<Re_{d_{p}}<10$). These large differences can be attributed to:

1. The pore diameter $d_{p}$ being insufficient to properly describe the foam structure.
2. The process of determining the permeability and inertial coefficient.

As discussed in De Schampheleire *et al.* [14], most manufacturers characterize their metal foam samples by reporting both the number of pores per (linear) inch (PPI) and the volumetric porosity ϕ. The porosity can be measured easily, and the same is true for the PPI value. However, because the cells are elongated in an anisotropic manner, there are at least two PPI values, depending on the direction of elongation. Furthermore, the flow behavior will also depend on the thickness of the struts. The effort to represent these three degrees of freedom with a single degree of freedom results in the large dispersion observed in the literature. When the foam is characterized with two cell diameters ($d_{1}$ and $d_{2}$ as illustrated in Figure 1) and the area in the middle of the strut ($A_{0}$), De Jaeger *et al.* [15] showed that the order of magnitude of dispersion on the experimental data becomes smaller than one.

Besides, manufacturers do not give any information on the microscopic nature of the foam itself. As illustrated in Figure 1, the thickness and the shape of the strut vary over its length. This distribution depends on the applied waxing process. Even the description of the foam with three degrees of freedom is therefore still not complete. Since the shape and the thickness distribution of the struts influence the pressure drop, this results in a residual dispersion of the results.

Most of the time, κ and β are determined experimentally. The pressure drop over the metal foam is determined and the quadratic fit is compared with Equation (2) to determine the permeability and inertial coefficient. A lot of issues arise from this method. First of all, the foam has to be so large that there are no wall effects [16] nor entrance/exit effects [17]. Furthermore, there are also large differences observed in κ and β depending on over which velocity range the quadratic fit is made. Innocentinni *et al.* [18] and Dukhan *et al.* [10] studied this and observed differences up to 75% in the value for permeability. Based on these results, Dukhan *et al.* [10] suggested the use of two permeability values depending on the flow regime: one for the Darcy regime and one for the Forchheimer regime.

In this work, the velocity dependence of the permeability (and inertial factor) will be investigated for a broad velocity range, covering both the Darcy regime and the Forchheimer regime.

## 2. Experimental Approach

**Figure 2:**
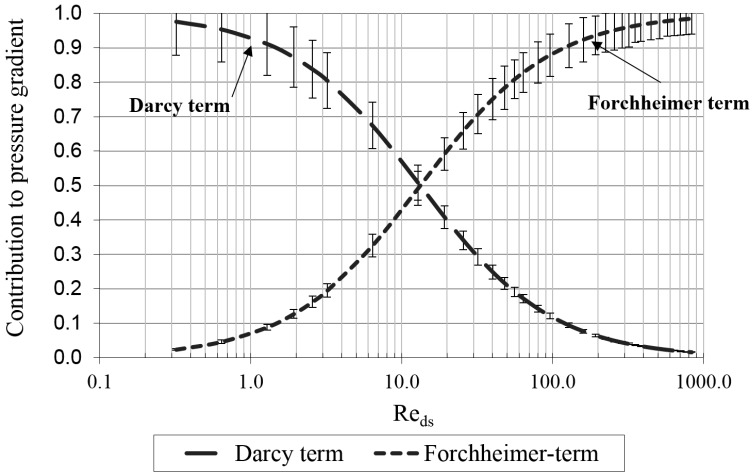
Contribution of the Darcy and Forchheimer term to the pressure gradient for a foam with following dimensions: $d_{1}:4.22\;mm,\;d_{2}:6.23\;mm$ and $A_{0}:0.0988\;mm^{2}$.

A first approach to investigating the effect of velocity on the permeability and the inertial factor is by performing experiments. Foam with a porosity of 93.2% ($d_{1}:4.22\;mm,\;d_{2}:6.23\;mm$ and $A_{0}:0.0988\;mm^{2}$) with a thickness of 40 mm is placed in a wind tunnel with a cross dimensional test section of 256 mm on 447 mm. The construction of the wind tunnel is explained in De Schampheleire *et al.* [19]. Pressure drop data for a velocity range between 0 and 26 m/s are gathered and fitted to a second order polynomial to determine κ and β. For this specific case these values were, respectively, $5.77\;\times 10^{-6}\;m^{2}$ and $118.59\frac{1}{m}$. With these values the Darcy term ($\frac{\mu }{\kappa }V$) and Forchheimer term ($\rho \beta V^{2}$) are determined and compared to the global pressure drop in Figure 2. The characteristic length for the Reynolds number is the average strut diameter: $d_{s}=4\left(1-\phi \right)/\sigma_{0}$ which can be interpreted as the diameter of a cylinder with a length equal to the total strut length and a volume equal to the solid phase volume [20]. The relative uncertainty is calculated through the error propagation rules as described in the textbook from Taylor [21]. It varies between 4.5% and 10%.

In the region of the low Reynolds numbers, corresponding to creeping flow, the Darcy term in Equation (2) is dominant and shows a nearly linear relation with the Reynolds number. The Forchheimer term becomes significant (more than 3% of the total pressure gradient) when the transition to the steady laminar regime occurs [22]. It indicates that inertial forces start to become significant. The share of the Forchheimer term increases significantly, until it contributes approximately 63% of the total pressure gradient [23]. The corresponding Reynolds number indicates the onset for the formation of regular vortex shedding from the struts, *i.e.*, transition to the unsteady laminar regime. The onset of transitional flow regime is clearly observed when the Forchheimer term contribution is approximately 83% [24]. For the transition to the turbulent flow regime, however, a study of Seguin *et al.* [24] states that the inception takes place when the Forchheimer contribution reaches 91% of the total pressure gradient.

The Darcy term accounts for the viscous drag, while the Forchheimer term corresponds to the form drag [10]. However, using numerical simulations, it is also possible to directly evaluate the viscous drag and the form drag separately and support this statement.

## 3. Numerical Approach

### 3.1. Equations and Calculation Method

Metal foam based on a polyurethane preform, for example, is hierarchically structured as shown by Lakes [25]. This means that the complete structure of the foam can be represented by its representative elementary volume (REV) and there exists a clear-cut length scale separation between the microscopically- and macroscopically-scaled physics [25]. The continuum and momentum equations can be averaged over that representative elementary volume, based on the volume averaging process as presented by Whitaker [26]. The REV consists of two phases, which Whitaker indicates by σ (solid) and ζ (fluid). An arbitrary quantity ψ of the ζ phase is indicated by $\psi_{\zeta }$. The intrinsic average of $\psi_{\zeta }$, evaluated at a location ${\vec{x}}_{c}$, is then given by $\langle \psi_{\zeta }\rangle \left({\vec{x}}_{c}\right)=\int_{V_{\zeta }}m\left({\vec{x}}_{c}-\vec{r}\right)\psi_{\zeta }\left(\vec{r}\right)\;dV$. Here, $m\left({\vec{x}}_{c}-\vec{r}\right)$ is a filter function, which is normalized on the REV volume $V_{m}$ and which is zero outside of the REV. This volume consists of both the σ and the ζ phases. Using Gray’s decomposition [27], the arbitrary quantity can be written as a function of the porosity ϕ, the intrinsic average and the microscopic scale deviation from the average: $\psi_{\zeta }=\phi \langle \psi_{\zeta }\rangle +{\tilde{\psi }}_{\zeta }$. Combining the volume averaged equations with the decomposition results in equations for the spatial deviation terms, given by Equations (5) and (6). The constraints for these equations are given in Whitaker [28].

(5) $$\nabla \cdot \tilde{\vec{v}}=0$$

(6) $$\rho \vec{v}\cdot \nabla \tilde{\vec{v}}=-\nabla \tilde{P}+\mu \nabla^{2}\tilde{\vec{v}}-\frac{1}{\phi }\int_{A_{fs}}m\left(\vec{x_{c}}-\vec{r}\right)\tilde{P}\left(\vec{r}\right){\vec{n}}_{fs}dA+\frac{1}{\phi }\int_{A_{fs}}m\left(\vec{x_{c}}-\vec{r}\right)\mu \nabla \tilde{\vec{v}}\left(\vec{r}\right)\cdot {\vec{n}}_{fs}dA$$

These deviation terms must also exhibit periodic behavior, which is mathematically expressed by Equations (7) and (8). The vector ${\vec{l}}_{n}$ expresses the translation of a REV over its dimensions in three directions. The spatial deviation terms in Equations (5) and (6) are mapped by Whitaker [28,29] on the intrinsically averaged velocity according to Equations (9) and (10). Equations (7) and (8) are based on Gray’s decomposition.

(7) $$\tilde{P}\left(\vec{r}+{\vec{l}}_{n}\right)=\tilde{P}\left(\vec{r}\right)$$

(8) $$\tilde{v}\left(\vec{r}+{\vec{l}}_{n}\right)=\tilde{v}\left(\vec{r}\right)$$

(9) $$\tilde{\vec{v}}=\overset{=}{M}\cdot \langle \vec{v}\rangle$$

(10) $$\tilde{P}=\mu \vec{m}\cdot \langle \vec{v}\rangle$$

Substituting these mapping functions in the closure problem and recognizing that $\langle \vec{v}\rangle$ is quasi-constant in a REV, the closure term problem can be re-written such that it requires solving for the two mapping functions $\overset{=}{M}$ and $\vec{m}$. An additional decomposition was done by Whitaker [28,29], which provides a theoretical framework for the derivation of the earlier-mentioned Darcy–Forchheimer equation. The first mapping function is fully related to the viscous drag, while the second mapping is related to the form drag effect. Therefore, the mapping functions need to be split and blended into new mapping functions in order to mimic the phenomenologically defined permeability and inertial loss factor [28,29].

The objective is to rewrite the average pressure gradient as a function of the average velocity. Inspired by the Darcy–Forchheimer relation (Equation (2)), a vectorial relation between the gradient of the average pressure and a second order polynomial function of the velocity can be imposed. A subscript asterisk is used to clearly distinguish the permeabilities from the Darcy–Forchheimer relation and the closure terms that are derived now, which will be called the direct formulation (Equation (11)). The link between the Darcian and superficial average velocity and the interfacial velocity is given by Equation (12).

(11) $$-\nabla {\langle P\rangle }^{i}=\mu \;{\overset{=}{\kappa }}_{*}^{-1}\cdot {\langle \vec{v}\rangle }^{s}+\rho {\overset{=}{\beta }}_{*}\cdot \left|{\langle \vec{v}\rangle }^{s}\right|{\langle \vec{v}\rangle }^{s}$$

(12) $${\langle \vec{v}\rangle }^{s}=\phi {\langle \vec{v}\rangle }^{i}$$

The momentum balance for the deviations can be rearranged into a similar form, resulting in Equation (13) [28]:

(13) $$-\nabla {\langle P\rangle }^{i}=-\int_{A_{fs}}m\left(\vec{x_{c}}-\vec{r}\right)\mu \nabla \tilde{\vec{v}}\left(\vec{r}\right)\cdot {\vec{n}}_{fs}dA+\frac{1}{\phi }\int_{A_{fs}}m\left(\vec{x_{c}}-\vec{r}\right)\tilde{P}\left(\vec{r}\right){\vec{n}}_{fs}dA$$

A term-to-term comparison between Equation (11) and Equation (13) allows deriving the direct formulation of the permeability and inertial coefficient [22]:

(14) $$\phi {\langle \vec{v}\rangle }^{i}\;\cdot {\overset{=}{\kappa }}_{*}^{-1}=-\int_{A_{fs}}m\left(\vec{x_{c}}-\vec{r}\right)\mu \nabla \tilde{\vec{v}}\left(\vec{r}\right)\cdot {\vec{n}}_{fs}dA$$

(15) $$\phi {\langle \vec{v}\rangle }^{i}\;\cdot {\overset{=}{\beta }}_{*}=\frac{1}{\rho \left|\phi {\langle \vec{v}\rangle }^{i}\right|}\int_{A_{fs}}m\left(\vec{x_{c}}-\vec{r}\right)\tilde{P}\left(\vec{r}\right){\vec{n}}_{fs}dA$$

Note that there is only one equation, which is used to determine two closure functions. Assuming that the real pressure drag behavior is in fact given by a second order polynomial, in the Darcy–Forchheimer equation (Equation (2)), the $\kappa^{-1}$ term is the first-order part and the β term is the second-order part. Here a different approach is followed, where the ${\overset{=}{\kappa }}_{*}^{-1}$ term is the viscous part and the ${\overset{=}{\beta }}_{*}$ term is the part due to pressure forces on the interface between the two phases. It is also apparent that the two terms need to be tensors, as the pressure drop can be expected to depend on the direction of the flow. This is due to the internal morphology of the metal foam, which is not isotropic, as can be seen in Figure 1.

**Figure 3:**
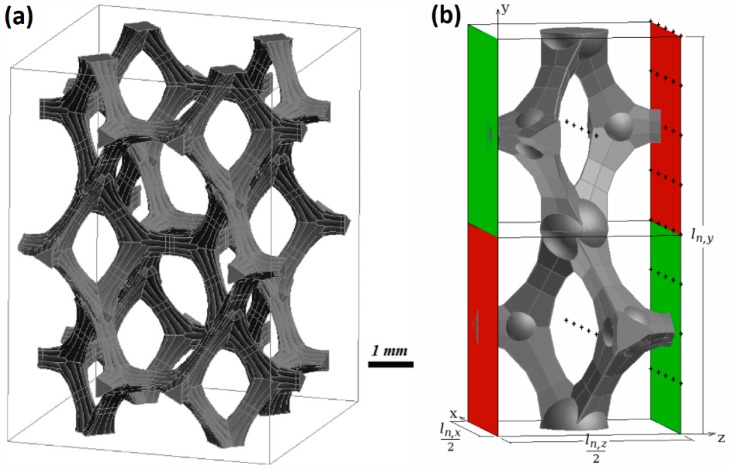
Illustration of the REV (**a**) and PUC (**b**) of the foam with dimensions: $d_{1}:4.22\;mm,\;d_{2}:6.23\;mm$ and $A_{0}:0.0988\;mm^{2}$.

Calculation of the viscous and pressure forces needs to be done over a REV. The derivation of the used volume is explained in the work of De Jaeger *et al.* [15]. As explained earlier, it is possible to derive a representative volume for the foam based on three parameters: $d_{1},d_{2}$ and $A_{0}$. The calculation method discussed previously will be applied for the foam mentioned in Section 2. The REV for this volume is determined by the model of De Jaeger *et al.* [15] and is shown in Figure 3a. In the PhD of De Jaeger [22] it is also shown that a quarter of this REV in the direction of the cell gives approximately the same results (within 12.5%) for pressure and viscous forces as the full REV, if the Reynolds number based on the specific cell diameter is lower than 200. This quarter REV is also called a periodic unit cell (PUC) and is shown in Figure 3b, where the cell is slightly modified by using small spheres in order to increase the quality of the computational grid.

Closer examination of the PUC reveals that a sub-volume bears additional periodicity. The green faces show geometrical periodicity, when a translation is performed in both the *z* and *y* direction. The red faces are also geometrically periodic in *z* direction, when a secondary translation in negative *y* direction is imposed. The same holds for the *x* direction. In the *y* direction, no secondary translation is needed to impose periodicity. Depending on the flow direction that is studied (see latter), different periodic boundary conditions (different Δp) were applied.

### 3.2. Calculation of Closure Terms

The microscopic quantities that are monitored are the viscous and form drag force densities and the intrinsically averaged velocity components. The former are denoted by ${\vec{f}}_{v}$ and ${\vec{f}}_{p}$, respectively, and computed in the CFD software as:

(16) $${\vec{f}}_{v}=\;-\;\frac{1}{V_{m}}\int_{A_{fs}}\mu \nabla \tilde{\vec{v}}\left(\vec{r}\right)\cdot {\vec{n}}_{fs}dA$$

(17) $${\vec{f}}_{p}=\frac{1}{V_{m}}\int_{A_{fs}}\tilde{P}\left(\vec{r}\right){\vec{n}}_{fs}dA$$

This corresponds to setting the filter $m\left(\vec{x_{c}}-\vec{r}\right)$ in Equations (6) and (13) equal to the reciprocal of the volume $V_{m}$ (covering both solid and fluid phase) when inside of the REV and equal to zero when outside of the REV. This corresponds to a block filter, but other filters could be chosen as well. The corresponding direct formulations of the permeability and inertial loss factor can be calculated through:

(18) $$\phi \langle \vec{v}\rangle \;\cdot {\overset{=}{\kappa }}_{*}^{-1}={\vec{f}}_{v}$$

(19) $$\phi \langle \vec{v}\rangle \;\cdot {\overset{=}{\beta }}_{*}=\frac{1}{\rho \left|\phi \langle \vec{v}\rangle \right|}{\vec{f}}_{p}$$

Both tensors are symmetrical and the non-diagonal components are zero [20,28,30]. For orthotropic media, Scheidegger [30] analyzed experimental data and revealed the symmetric behavior. This was later proven by Whitaker [28]. Thus, when permeability is determined along the principal directions, only the three diagonal components need to be determined. For the inertial loss factor though, symmetry is not guaranteed. Magnico [20], however, investigated this factor for shear-deformed open-cell nickel foams and found that the eigenvectors were nearly orthogonal and they followed the shear angles. It led to the conclusion that the inertial loss factor of foams could practically be considered symmetrical. Furthermore, the permeability and inertial coefficient along the *z*-direction is the same as the one along the *x*-direction ($\kappa_{*,xx}=\kappa_{*,zz}$ and $\beta_{*,xx}=\beta_{*,zz}$ with *x*, *y* and *z* as indicated in Figure 3b). There are therefore just two unknown components in each tensor, which can be obtained imposing flow in two different directions. Pressure gradients of different magnitudes are imposed, once in the *x* direction and once in the *y* direction. The calculations are done using a commercial CFD software package. The convective terms are discretized using a second-order upwind scheme and a coupled pressure-velocity scheme is used. No turbulence model is used. All residuals have to be lower than $10^{-6}$ before accepting the solution.

Shear stress calculation in laminar flow (${\vec{f}}_{v}$) requires a sufficiently fine mesh at the boundary layer to accurately resolve the gradient. In order to be certain that changing the size of the cells of the computational grid does not influence the results for ${\overset{=}{\kappa }}_{*}^{}$ and ${\overset{=}{\beta }}_{*}$, a grid discretization study is performed. In this work the Roache’s grid convergence index (GCI) is used to estimate of the grid discretization error [31,32]. Three different grid sizes are tested each with a 10% refinement of all cells in each direction. For the finest mesh, the first boundary layer cell was 4 µm thick. The growth ratio was taken as 1.1 and a maximum size cell of 60 µm is imposed. For the PUC reported in Figure 3b this leads to a computational domain of 10.5 million cells. In Table 1 the GCI for the finest grid is reported. Even for this fine grid, the relative uncertainty on $\kappa_{*,yy}$ is quite high (15.4%). However, the uncertainties are acceptable in comparison to the experimental results, where uncertainties over one order of magnitude are reported (see Bonnet *et al.* [13]).

### 3.3. Results in Laminar Regime

**Figure 4:**
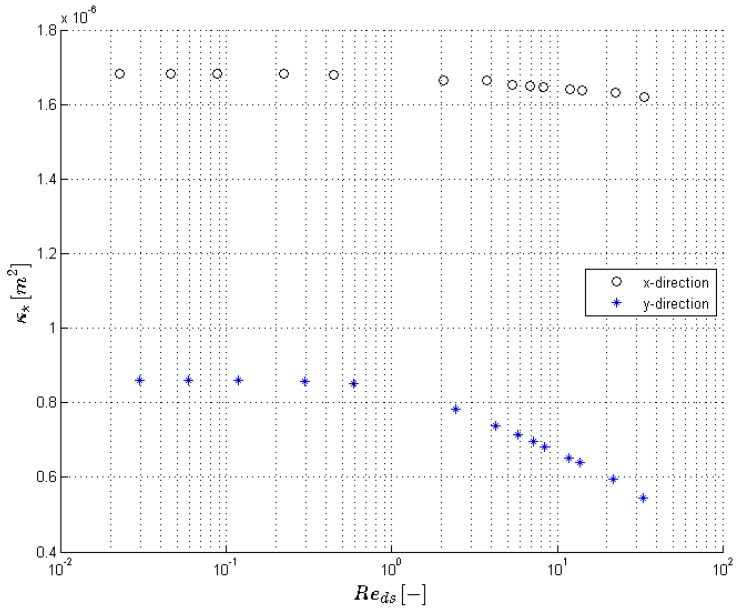
The permeability in the *x* and *y* direction ($\kappa_{*,xx}$ and $\kappa_{*,yy}$) determined through numerical calculations plotted against the Reynolds number.

**Figure 5:**
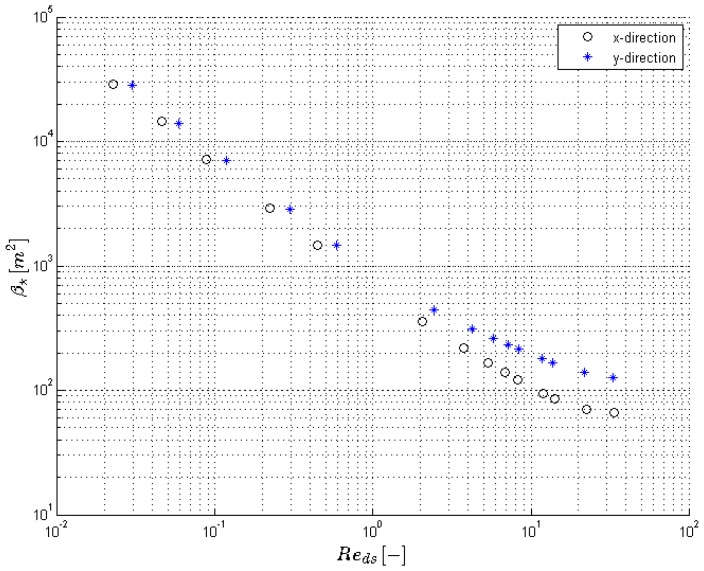
The inertial coefficient in the *x* and *y* direction ($\beta_{*,xx}$ and $\beta_{*,yy}$) determined through numerical calculations plotted against the Reynolds number.

The results for the different pressure gradients that were simulated are reported in Table 2 and Figure 4 and Figure 5. The focus of this paper is low velocities: only steady calculations were performed.

The permeability in both directions remains constant for $Re_{d_{s}}<0.25$, see Figure 4 and Table 2. The flow regime here is creeping flow. For higher Reynolds numbers, the viscous force (shear stress) will start to increase and by observing the trend, it can be stated that the viscous force increases slightly more than linearly with the Darcian velocity. According to Equation (18), this results in a decrease of the permeability.

The inertial coefficient in the direct formulation is defined as the ratio of the (volume averaged) pressure force to the kinetic energy of the fluid (see Equation (15)). Upstream of a strut, there is a stagnation zone where kinetic energy descends to zero, which results in a region with high pressure. Downstream, a distinction has to be made between flow regimes with or without recirculation regions in the wakes behind struts. In case of no recirculation ($Re_{d_{s}}<10$, see Figure 5), the inertial loss factor decreases with increasing Reynolds number. This means that the pressure force increases at a rate lower than the average kinetic energy in the flow domain. For higher Reynolds numbers, it is expected that the increment of pressure force will be balanced by the increase of velocity. This will be again characterized by a nearly constant inertial coefficient. This can be again observed in Table 2 for the Reynolds numbers in laminar regime.

However, recalling the discussion in Section 2, a well-known interpretation of the Reynolds number is that for low velocities ($Re_{d_{s}}<1$), one should expect a primarily viscous flow. However, looking to the influence of the pressure force compared to the viscous force, the pressure influence cannot be neglected.

To provide more detail, Table 3 reports the viscous and pressure forces over the simulated range of Reynolds numbers in the *x*-direction. Similar results hold for the *y*-direction. Next to both the viscous and pressure forces, the influence of the viscous force to the total force ($\frac{f_{v,x}}{f_{v,x}+f_{p,x}}$) is also reported in Table 3. The maximum influence of the viscous forces is only 32%. Although both forces increase with increasing Reynolds numbers, the relative influence of the viscous forces rapidly decreases. As expected, for high Reynolds numbers the inertial contribution to the drag becomes constant.

**Figure 6:**
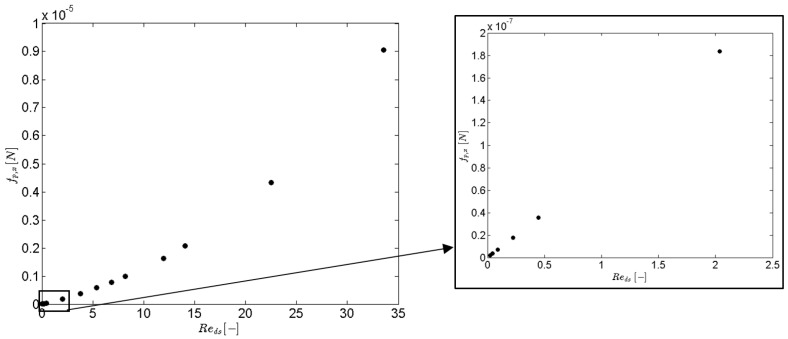
The pressure force in the *x*-direction is plotted against the Reynolds number.

Furthermore, from Figure 6 and Table 3 it is also clear that the pressure force varies linearly with velocity for small Reynolds numbers ($Re_{d_{s}}<2$). For this velocity range, $\beta_{*,xx}$ and $\beta_{*,yy}$ can be written as a constant value divided by the velocity (see Equation (15) with $\vec{f_{p}}\;\sim \;\vec{v})$. In the case of the studied foam, $\beta_{*,xx}=19.76/v_{x}$ for $Re_{d_{s}}<2$ and $\beta_{*,yy}=25.68/v_{y}$ for $Re_{d_{s}}<2$.

### 3.4. Discussion on the Darcy Equation

The large influence of pressure forces at low velocities can be explained by looking to the theory of Stokes flow. Rewriting the momentum equations in dimensionless form, it can be shown that for very low Reynolds numbers, the material derivative of the velocity can be neglected. Equivalently, this means that inertial effects are neglected. However, it is important to note that the pressure gradient can still be significant in comparison to the viscous term. Only neglecting the inertial term but keeping the pressure term results in the so-called Stokes equation [33] (Equation (20)). In this equation, the pressure is made dimensionless with respect to μU/L, where U is the free stream velocity and *L* is a characteristic length scale.

(20) $$\nabla p-\mu \;\nabla^{2}\vec{v}=0$$

For a 3D sphere, there exists an exact analytical solution for the drag. With *a*, the radius of the sphere and *V*, the unidirectional incoming velocity, it is given by Equation (17) [33]:

(21) $$D=\underset{pressure}{\underbrace{3\pi \mu aV\int_{0}^{\pi }cos^{2}\theta sin\theta \;d\theta }}+\underset{viscous}{\underbrace{3\pi \mu aV\int_{0}^{\pi }sin^{3}\theta d\theta }}=2\pi \mu aV+4\pi \mu aV=6\pi \mu aV$$

In the case of a 3D sphere, one-third of the drag is due to pressure forces and two-thirds is due to viscous forces. This is also verified with the CFD software used in this work. However, from Table 3, even higher influences of the pressure forces are observed in the case of flow around metal foam. This is because the struts themselves do not have the shape of a sphere. They can however be approximated by flow around a cylinder or a triangular prism. Furthermore, the flow is not around a single strut, but around a staggered array of struts, which has different flow characteristics.

**Figure 7:**
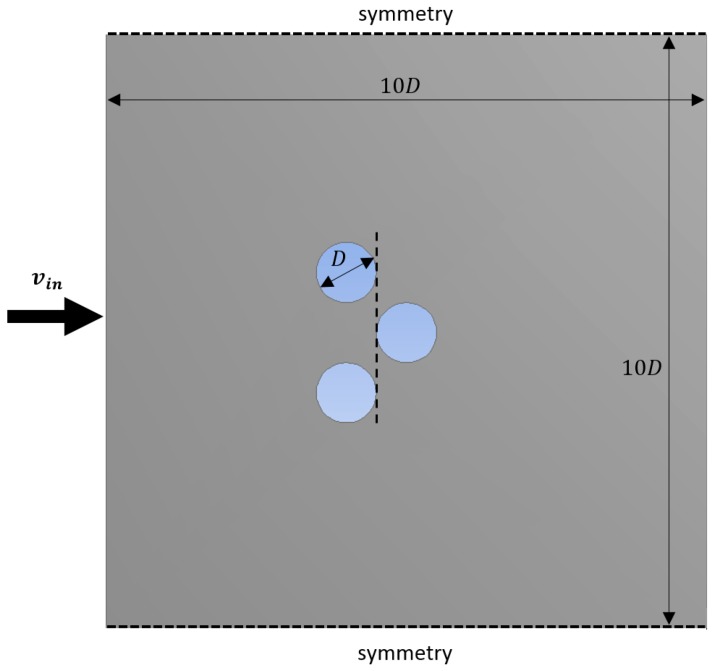
Illustration of the boundary conditions for the staggered case with circles.

**Figure 8:**
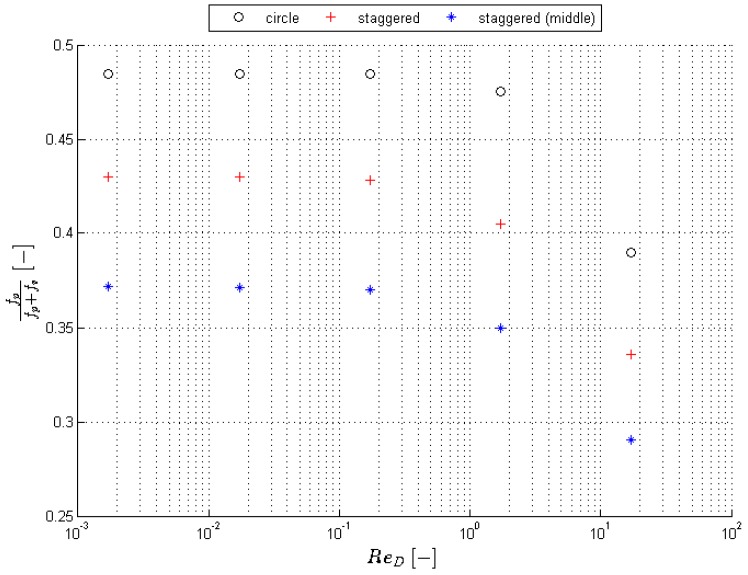
Illustration of the influence against the velocity of the viscous forces to the total forces acting on the surface of the foam.

To illustrate the influence of the pressure forces, some additional calculations are performed on the following geometries: (1) a standalone circle; and (2) three circles in staggered layout (see Figure 7). For these simulations, the solution techniques and discretization of the geometry is done exactly the same as in case of the finest mesh discussed above. Of course, instead of using the volume-averaged equations, the classical Navier–Stokes equations are used here. If the strut diameter is *D*, the surroundings are 10*D* (see Figure 7). The influence of the viscous force on the total force is reported in Figure 8 for a single circle and three circles placed in a staggered configuration. As can be seen, the staggered layout of the circles results in a lower relative influence of the viscous forces. Furthermore, the middle circle of the staggered layout experiences an even lower influence of viscous forces, namely only 37%. These observations are consistent with the observations from Table 3: the contributions of the viscous forces are very low in a real foam.

As illustrated in Figure 8, the pressure influences are not negligible at low velocities. Furthermore, from Figure 6 it is clear that the pressure drop for $Re_{d_{s}}<2$ varies linearly with the velocity. Thus, the pressure drop over the foam can be written as a combination of viscous and pressure forces (over a microscopic element):

(22) $$\frac{dp}{dx}=\underset{viscous}{\underbrace{\frac{\mu }{\kappa_{vis}}v}}+\underset{pressure}{\underbrace{C\;v}}$$

Note again that this equation is different compared to the classical Darcy equation where the permeability is a combination of viscous and inertial influences. *C* in Equation (22) is a constant parameter representing the influence of the pressure drag on the pressure drop. In order to rewrite Equation (22) to the generally know Darcy equation, *C* should vary linearly with the molecular viscosity, such that it can be written as $C=\frac{\mu }{\kappa_{inertial}}$.

**Figure 9:**
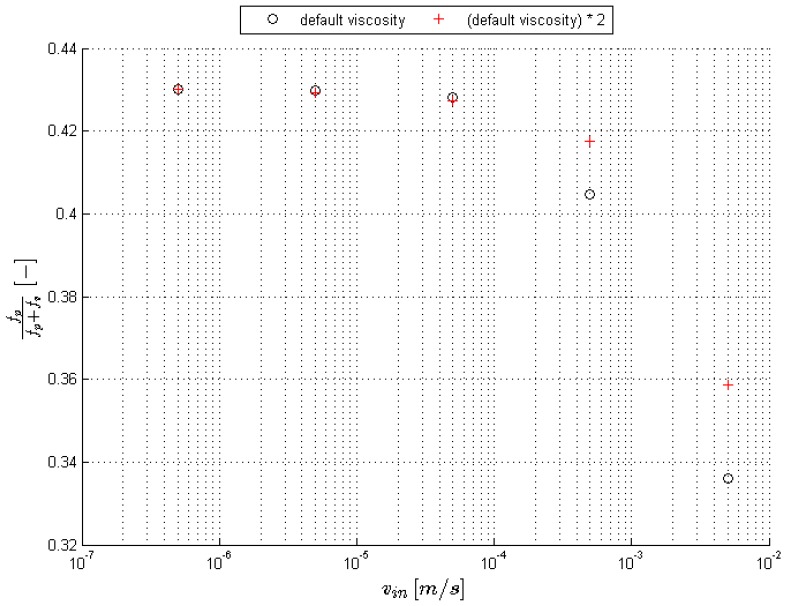
Illustration of the influence against the velocity of the viscous forces to the total forces acting on the surface of the foam for the staggered circle layout and two different viscosities.

To investigate this, the simulations of the staggered layout are repeated but with an increase of the fluid viscosity with a factor of 2. Increasing the viscosity will also increase the viscous forces with the same factor, since there is a linear relation between both. From the dimensionless relation for the pressure in the Stokes flow, it is expected that the pressure gradient will also get scaled linearly with the viscosity. The results are depicted in Figure 9. It is confirmed that in creeping flow where inertial effects are negligible and Stokes flow is valid, for $Re_{d_{s}}<2$, the pressure drag indeed varies linearly with the viscosity. This means that the Darcy law is still valid, see Equation (23), with $\kappa_{classical}=\kappa$ as reported in open literature. However, one needs to be careful with the interpretation of the Darcy equation. In Dukhan *et al.* [10], it was mentioned that the Darcy equation was representing the viscous drag, but the permeability as reported in the Darcy equation is really a combination of a viscous and pressure drag component.

(23) $$\frac{dp}{dx}=\frac{\mu }{\kappa_{vis}}v+\frac{\mu }{\kappa_{pressure}}v=\frac{\mu }{\kappa_{classical}}v$$

The direct formulation of the permeability and the inertial coefficient shows velocity-dependent behavior, which is due to two reasons. Firstly, the linear term of the pressure drop is not purely due to the shear stress, since the pressure drag also exhibits linear behavior for low Reynolds numbers. Similarly, it is not really correct to lump the pressure drag into the inertial term for these low velocities, as it really has a linear behavior and not a quadratic behavior. Secondly, the real pressure drag *versus* velocity behavior is not exactly given by a second order polynomial, which results in velocity-dependent values for the permeability and the inertial coefficient even in the phenomenological approach of the Darcy law.

## 4. Conclusions

This work has pointed out that there is another way to calculate permeability and inertial coefficient. Based on a numerical approach, both closure terms are calculated depending on, respectively, the viscous and pressure forces acting on a representative elementary volume of the studied open-cell foam. It was shown that in the creeping flow regime, the linear term in the Darcy law was due to both pressure forces and viscous forces. Furthermore, for creeping flow and based on the Stokes equation, it is shown that this pressure force influence is more important than the viscous contribution with a ratio of 70%/30%. Finally, in the volume averaging theory, the pressure forces are associated with inertial effects (quadratic in function of the velocity), which is, strictly speaking, not valid for the creeping flow regime. This results in the inertial coefficient in the direct formulation going to infinity as the velocity goes to zero, varying as the reciprocal of the flow velocity.

## Figures and Tables

**Table 1:** Determination of grid discretization error for a pressure gradient of 100 Pa over the PUC.

	Coarse Mesh (Start Size: 5 µm)	Finer Mesh (Start Size: 4.5 µm)	Finest Mesh (Start Size: 4 µm)	$\Delta_{coarse-finest}$	$GCI_{finest\;grid}$
$\kappa_{*,xx}$	$1.584\;\times \;10^{-6}\;m^{2}$	$1.610\;\times \;10^{-6}\;m^{2}$	$1.632\;\times \;10^{-6}\;m^{2}$	3%	7.2%
$\kappa_{*,yy}$	$6.318\;\times \;10^{-7}\;m^{2}$	$6.527\;\times \;10^{-7}\;m^{2}$	$6.700\;\times \;10^{-7}\;m^{2}$	6%	15.4%
$\beta_{*,xx}$	$72.36\frac{1}{m}$	$72.66\frac{1}{m}$	$73.74\frac{1}{m}$	1.9%	8.7%
$\beta_{*,yy}$	$142.75\frac{1}{m}$	$143.70\frac{1}{m}$	$144.54\frac{1}{m}$	1.2%	3.44%

**Table 2:** Results for the permeability and inertial coefficient based on the numerical calculation method.

$Re_{d_{s},\;x}$	$Re_{d_{s},\;y}$	$\kappa_{*,xx}$ (m^2^)	$\kappa_{*,yy}$ (m^2^)	$\beta_{*,xx}$ (1/m)	$\beta_{*,yy}$ (1/m)
0.02244	0.02982	1.682 × 10^−6^	8.600 × 10^−7^	28,744	28,062
0.04581	0.05889	1.682 × 10^−6^	8.600 × 10^−7^	14,374	14,037
0.08834	0.1178	1.682 × 10^−6^	8.600 × 10^−7^	7191.5	7029.9
0.2225	0.2977	1.681 × 10^−6^	8.580 × 10^−7^	2888.6	2843
0.4450	0.5857	1.680 × 10^−6^	8.510 × 10^−7^	1464.6	1472.7
2.0384	2.4474	1.664 × 10^−6^	7.820 × 10^−7^	360.79	444.38
3.7659	4.2338	1.663 × 10^−6^	7.380 × 10^−7^	218.26	311.63
5.3462	5.7618	1.654 × 10^−6^	7.130 × 10^−7^	165.99	260.38
6.8218	7.1327	1.649 × 10^−6^	6.950 × 10^−7^	138.24	231.67
8.2059	8.3926	1.645 × 10^−6^	6.810 × 10^−7^	120.93	212.72
11.9227	11.7460	1.639 × 10^−6^	6.530 × 10^−7^	94.22	180.00
14.0919	13.7320	1.637 × 10^−6^	6.400 × 10^−7^	85.39	167.31
22.5039	21.8332	1.632 × 10^−6^	5.960 × 10^−7^	69.85	138.94
33.5792	33.0721	1.620 × 10^−6^	5.430 × 10^−7^	65.65	126.75

**Table 3:** Results for the pressure and viscous forces acting on the PUC for different Reynolds numbers.

$Re_{d_{s},\;x}$	$f_{p,x}$ (N)	$f_{v,x}$ (N)	$\frac{f_{v,x}}{f_{v,x}+f_{p,x}}$
0.02243	1.77 × 10^−9^	8.14 × 10^−10^	0.315
0.0458	3.54 × 10^−9^	1.63 × 10^−9^	0.315
0.0883	7.08 × 10^−9^	3.26 × 10^−9^	0.315
0.2225	1.77 × 10^−8^	8.13 × 10^−9^	0.315
0.4450	3.55 × 10^−8^	1.62 × 10^−8^	0.313
2.0384	1.84 × 10^−7^	7.48 × 10^−8^	0.290
3.7659	3.79 × 10^−7^	1.38 × 10^−7^	0.267
5.3462	5.8 × 10^−7^	1.95 × 10^−7^	0.251
6.8219	7.87 × 10^−7^	2.47 × 10^−7^	0.239
8.2059	9.96 × 10^−7^	2.96 × 10^−7^	0.229
11.9227	1.64 × 10^−6^	4.29 × 10^−7^	0.208
14.0920	2.07 × 10^−6^	5.1 × 10^−7^	0.197
22.5040	4.33 × 10^−6^	8.42 × 10^−7^	0.163
33.5792	9.05 × 10^−6^	1.28 × 10^−6^	0.124
